# 3-Benzoyl-1-(2-meth­oxy­phen­yl)thio­urea

**DOI:** 10.1107/S160053681204456X

**Published:** 2012-11-03

**Authors:** N. Selvakumaran, M. Mary Sheeba, R. Karvembu, Seik Weng Ng, Edward R. T. Tiekink

**Affiliations:** a Department of Chemistry, National Institute of Technology, Tiruchirappalli 620 015, India; b Department of Chemistry, University of Malaya, 50603 Kuala Lumpur, Malaysia; c Chemistry Department, Faculty of Science, King Abdulaziz University, PO Box 80203 Jeddah, Saudi Arabia

## Abstract

In the title compound, C_15_H_14_N_2_O_2_S, the central C_2_N_2_OS moiety is planar (r.m.s. deviation of fitted atoms = 0.0336 Å). This is ascribed to the formation of an *S*(6) loop stabilized by an intra­molecular N—H⋯O hydrogen bond; additional intramolecular N—H⋯O and C—H⋯S contacts are also noted. The dihedral angles between the central unit and the phenyl and benzene rings are 23.79 (7) and 29.52 (5)°, respectively. The thione S and ketone O atoms are mutually *anti*, as are the N—H H atoms; the O atoms lie to the same side of the mol­ecule. Centrosymmetric eight-membered {⋯HNC=S}_2_ synthons feature in the crystal packing. The resulting inversion dimers stack along the *a* axis and are connected into a three-dimensional structure by C—H⋯O and C—H⋯π inter­actions.

## Related literature

For complexation of *N*-benzoyl-*N*′-aryl­thio­urea derivatives to transition metals, see: Selvakumaran *et al.* (2011 ▶). For the structure of the unsubstituted parent compound, see: Yamin & Yusof (2003 ▶).
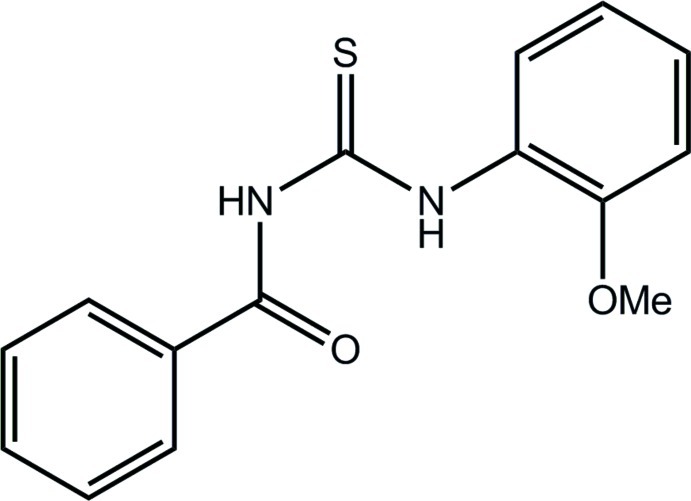


## Experimental

### 

#### Crystal data

C_15_H_14_N_2_O_2_S
*M* *_r_* = 286.34
Monoclinic, 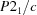

*a* = 5.9358 (1) Å
*b* = 25.6916 (4) Å
*c* = 9.0535 (1) Å
β = 92.065 (1)°
*V* = 1379.76 (4) Å^3^
*Z* = 4
Cu *K*α radiation
μ = 2.11 mm^−1^
*T* = 100 K
0.30 × 0.25 × 0.20 mm

#### Data collection

Agilent SuperNova Dual diffractometer with an Atlas detector
Absorption correction: multi-scan (*CrysAlis PRO*; Agilent, 2012 ▶) *T*_min_ = 0.467, *T*_max_ = 1.000
5143 measured reflections
2721 independent reflections
2505 reflections with *I* > 2σ(*I*)
*R*_int_ = 0.016

#### Refinement

*R*[*F* ^2^ > 2σ(*F* ^2^)] = 0.034
*wR*(*F* ^2^) = 0.097
*S* = 1.04
2721 reflections
189 parameters
H atoms treated by a mixture of independent and constrained refinement
Δρ_max_ = 0.27 e Å^−3^
Δρ_min_ = −0.40 e Å^−3^

### 

Data collection: *CrysAlis PRO* (Agilent, 2012 ▶); cell refinement: *CrysAlis PRO*; data reduction: *CrysAlis PRO*; program(s) used to solve structure: *SHELXS97* (Sheldrick, 2008 ▶); program(s) used to refine structure: *SHELXL97* (Sheldrick, 2008 ▶); molecular graphics: *ORTEP-3 for Windows* (Farrugia, 1997 ▶) and *DIAMOND* (Brandenburg, 2006 ▶); software used to prepare material for publication: *publCIF* (Westrip, 2010 ▶).

## Supplementary Material

Crystal structure: contains datablock(s) global, I. DOI: 10.1107/S160053681204456X/su2519sup1.cif

Structure factors: contains datablock(s) I. DOI: 10.1107/S160053681204456X/su2519Isup2.hkl

Supplementary material file. DOI: 10.1107/S160053681204456X/su2519Isup3.cml

Additional supplementary materials:  crystallographic information; 3D view; checkCIF report

## Figures and Tables

**Table 1:** Hydrogen-bond geometry (Å, °) *Cg*1 is the centroid of the C9–C14 benzene ring.

*D*—H⋯*A*	*D*—H	H⋯*A*	*D*⋯*A*	*D*—H⋯*A*
N2—H2*n*⋯O1	0.905 (18)	1.867 (18)	2.6316 (15)	141.0 (16)
C10—H10⋯S1	0.95	2.68	3.2241 (13)	117
N2—H2*n*⋯O2	0.90 (2)	2.231 (19)	2.5819 (15)	102.5 (14)
N1—H1*n*⋯S1^i^	0.902 (18)	2.636 (18)	3.4976 (12)	160.1 (15)
C15—H15*B*⋯O1^ii^	0.98	2.57	3.4273 (19)	146
C15—H15*C*⋯*Cg*1^iii^	0.98	2.81	3.6248 (17)	141

Symmetry codes: (i) 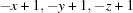; (ii) 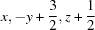; (iii) 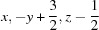.

**Fig. 1.**
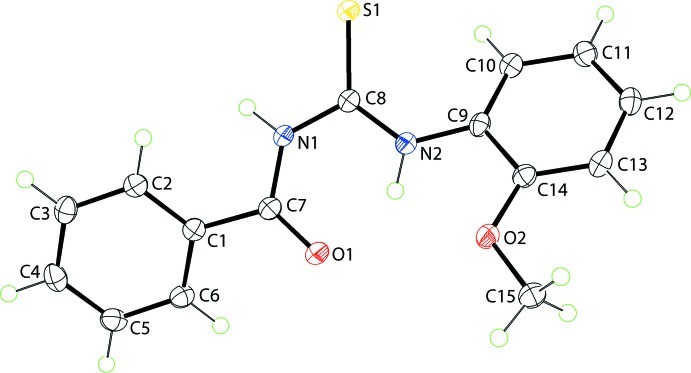
Molecular structure of the title molecule, showing the atom-labelling. Displacement ellipsoids are drawn at the 50% probability level.

**Fig. 2.**
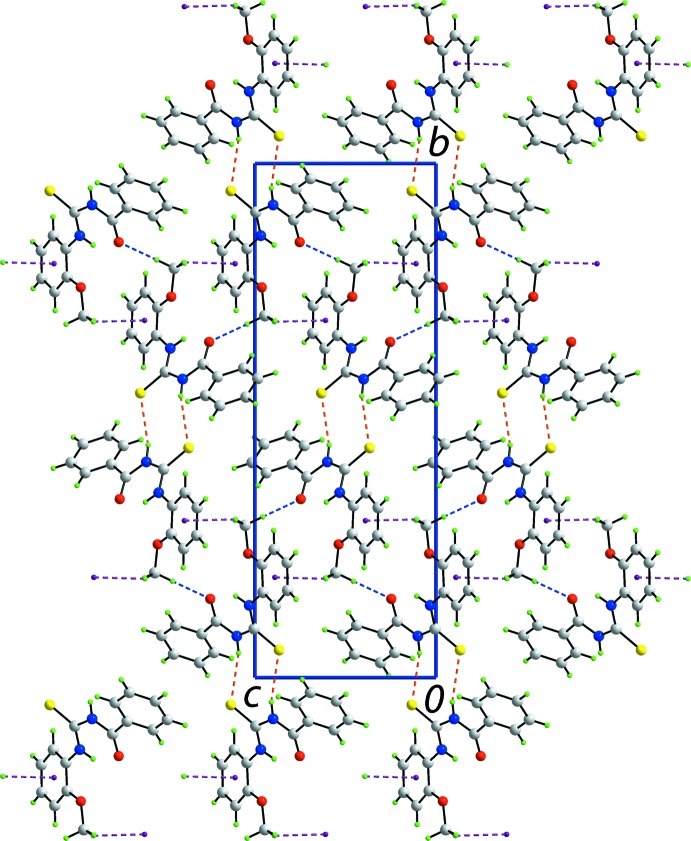
A view of the unit-cell contents of the title compound in projection down the a axis. The N—H···S, C—H···O and C—H···π interactions are shown as orange, blue and purple dashed lines, respectively.

## supplementary crystallographic information

### Comment

*N*-Benzoyl-*N*'-arylthiourea derivatives, of which the title compound is an example are versatile ligands with coordination possible *via* the S, O or N atom, and various combinations of these. The title compound was prepared in connection with on-going studies involving complexation of *N*-benzoyl-*N*'-arylthiourea derivatives to transition metals, *e.g*. Pd^II^ (Selvakumaran *et al.*, 2011).

In the title compound, Fig. 1, the thione and ketone atoms are *anti* with respect to each other. Similarly, the N—H H-atoms are *anti* to each other, and the oxygen atoms lie to the same side of the molecule. The central chromophore is planar with a r.m.s. deviation of 0.0336 Å [maximum deviations of 0.0449 (5) Å for S1, and -0.0460 (9) Å for N1] owing to the presence of an intramolecular N—H···O hydrogen bond which closes an *S*(6) loop (Table 1). Weaker intramolecular N—H···O and C—H···S contacts are also noted (Table 1). Despite this, a significant twist is evident in the molecule as manifested in the dihedral angle of 53.06 (5)° between the six-membered rings. The relative orientation of the atoms and deviations from planarity described above mimic those reported for the unsubstituted parent compound, PhC(═ O)N(H)C(═ S)N(H)Ph where the dihedral angle between the phenyl rings is 33.26 (6)° (Yamin & Yusof, 2003).

The most significant interaction in the crystal packing is the formation of centrosymmetric eight-membered {···HNC=S}_2_ synthons owing to the presence of N—H···S hydrogen bonds (Table ). These inversion dimers stack along the *a* axis and are connected into a three-dimensional architecture by C—H···O and C—H···π interactions (Fig. 2 and Table 1).

### Experimental

A solution of benzoyl chloride (0.005 mol, 0.7029 g) in acetone (30 ml) was added drop wise to a suspension of potassium thiocyanate (0.005 mol, 0.4859 g) in anhydrous acetone (30 ml). The reaction mixture was heated under reflux for 45 minutes and then cooled to room temperature. A solution of substituted 2-methoxyaniline (0.005 mol, 0.6158 g). in acetone (30 ml) was added and the resulting mixture was stirred for 2 h. Hydrochloric acid (0.1 N, 300 ml) was added and resulting solid was filtered, washed with water and dried *in vacuo*. The resulting solid product was recrystallized from ethanol/dichloromethane (1:2 ratio) solution. Yield: 87%, *M*. pt: 409 K. Anal. Calcd. for C_15_H_14_N_2_O_2_S (%): C, 62.9; H, 4.9; N, 9.8; Found: C, 63.1; H, 5.1; N, 9.6. Spectroscopic data for the title compound are given in the archived CIF.

### Refinement

Carbon-bound H-atoms were placed in calculated positions [C—H = 0.95 to 0.98 Å, *U*_iso_(H) = 1.2U_eq_(C) or 1.5*U*_eq_(C_methyl_)] and were included in the refinement in the riding model approximation. The N-bound H-atoms were refined freely.

### Crystal data

**Table:**

C_15_H_14_N_2_O_2_S	*F*(000) = 600
*M**_r_* = 286.34	*D*_x_ = 1.378 Mg m^−^^3^
Monoclinic, *P*2_1_/*c*	Cu *K*α radiation, λ = 1.54184 Å
Hall symbol: -P 2ybc	Cell parameters from 3350 reflections
*a* = 5.9358 (1) Å	θ = 3.4–74.2°
*b* = 25.6916 (4) Å	µ = 2.11 mm^−^^1^
*c* = 9.0535 (1) Å	*T* = 100 K
β = 92.065 (1)°	Block, colourless
*V* = 1379.76 (4) Å^3^	0.30 × 0.25 × 0.20 mm
*Z* = 4	

### Data collection

**Table:**

Agilent SuperNova Dual diffractometer with an Atlas detector	2721 independent reflections
Radiation source: SuperNova (Cu) X-ray Source	2505 reflections with *I* > 2σ(*I*)
Mirror monochromator	*R*_int_ = 0.016
Detector resolution: 10.4041 pixels mm^-1^	θ_max_ = 74.4°, θ_min_ = 3.4°
ω scan	*h* = −7→6
Absorption correction: multi-scan (*CrysAlis PRO*; Agilent, 2012)	*k* = −31→12
*T*_min_ = 0.467, *T*_max_ = 1.000	*l* = −11→10
5143 measured reflections	

### Refinement

**Table:**

Refinement on *F*^2^	Primary atom site location: structure-invariant direct methods
Least-squares matrix: full	Secondary atom site location: difference Fourier map
*R*[*F*^2^ > 2σ(*F*^2^)] = 0.034	Hydrogen site location: inferred from neighbouring sites
*wR*(*F*^2^) = 0.097	H atoms treated by a mixture of independent and constrained refinement
*S* = 1.04	*w* = 1/[σ^2^(*F*_o_^2^) + (0.0671*P*)^2^ + 0.2141*P*] where *P* = (*F*_o_^2^ + 2*F*_c_^2^)/3
2721 reflections	(Δ/σ)_max_ = 0.002
189 parameters	Δρ_max_ = 0.27 e Å^−^^3^
0 restraints	Δρ_min_ = −0.40 e Å^−^^3^

### Special details

**Table:**

Experimental. Spectroscopic data for the title compound:^1^H NMR (400 MHz, CDCl_3_, p.p.m.): 3.96 (s, 3H, OCH3); 6.96–7.91 (m, 8H); 8.76 (dd, J = 8.0 Hz & 1.6 Hz, 1H); 9.11 (s, 1H, thiourea NH); 12.85 (s, 1H, amide NH). ^13^C NMR (400 MHz, CDCl_3_, p.p.m.): 56.1; 110.6; 120.2; 123.0; 126.8; 127.2; 127.5; 129.1; 131.1; 133.1; 150.7; 166.5; 176.7. F T—IR (KBr, cm^-1^): 3270 ν(amide N—H), 3014 ν(thiourea N—H), 1672 ν(C═O), 1240 ν(C═S).
Geometry. All e.s.d.'s (except the e.s.d. in the dihedral angle between two l.s. planes) are estimated using the full covariance matrix. The cell e.s.d.'s are taken into account individually in the estimation of e.s.d.'s in distances, angles and torsion angles; correlations between e.s.d.'s in cell parameters are only used when they are defined by crystal symmetry. An approximate (isotropic) treatment of cell e.s.d.'s is used for estimating e.s.d.'s involving l.s. planes.
Refinement. Refinement of *F*^2^ against ALL reflections. The weighted *R*-factor *wR* and goodness of fit *S* are based on *F*^2^, conventional *R*-factors *R* are based on *F*, with *F* set to zero for negative *F*^2^. The threshold expression of *F*^2^ > σ(*F*^2^) is used only for calculating *R*-factors(gt) *etc*. and is not relevant to the choice of reflections for refinement. *R*-factors based on *F*^2^ are statistically about twice as large as those based on *F*, and *R*- factors based on ALL data will be even larger.

### Fractional atomic coordinates and isotropic or equivalent isotropic displacement parameters (Å^2^)

**Table:**

	*x*	*y*	*z*	*U*_iso_*/*U*_eq_	
S1	0.34254 (5)	0.553335 (12)	0.63331 (3)	0.02037 (13)	
O1	0.58134 (17)	0.65291 (4)	0.25290 (11)	0.0228 (2)	
O2	0.30344 (16)	0.74084 (4)	0.46245 (11)	0.0221 (2)	
N1	0.56616 (18)	0.58059 (4)	0.39976 (12)	0.0168 (2)	
H1n	0.617 (3)	0.5477 (7)	0.410 (2)	0.025 (4)*	
N2	0.29972 (18)	0.64037 (4)	0.46801 (12)	0.0173 (2)	
H2n	0.361 (3)	0.6586 (7)	0.394 (2)	0.034 (5)*	
C1	0.8406 (2)	0.58513 (5)	0.20667 (14)	0.0176 (3)	
C2	0.9874 (2)	0.54873 (5)	0.27253 (15)	0.0190 (3)	
H2	0.9654	0.5374	0.3709	0.023*	
C3	1.1658 (2)	0.52905 (5)	0.19390 (16)	0.0217 (3)	
H3	1.2654	0.5042	0.2385	0.026*	
C4	1.1982 (2)	0.54569 (5)	0.05014 (16)	0.0236 (3)	
H4	1.3204	0.5323	−0.0032	0.028*	
C5	1.0521 (2)	0.58180 (5)	−0.01569 (15)	0.0231 (3)	
H5	1.0740	0.5929	−0.1142	0.028*	
C6	0.8744 (2)	0.60160 (5)	0.06219 (15)	0.0210 (3)	
H6	0.7754	0.6265	0.0171	0.025*	
C7	0.6527 (2)	0.60957 (5)	0.28644 (14)	0.0178 (3)	
C8	0.3977 (2)	0.59455 (5)	0.49537 (14)	0.0165 (3)	
C9	0.1267 (2)	0.66553 (5)	0.54421 (14)	0.0173 (3)	
C10	−0.0476 (2)	0.64063 (5)	0.61365 (14)	0.0191 (3)	
H10	−0.0534	0.6037	0.6158	0.023*	
C11	−0.2144 (2)	0.66955 (6)	0.68032 (15)	0.0213 (3)	
H11	−0.3336	0.6523	0.7276	0.026*	
C12	−0.2069 (2)	0.72356 (6)	0.67787 (16)	0.0228 (3)	
H12	−0.3192	0.7431	0.7253	0.027*	
C13	−0.0354 (2)	0.74907 (5)	0.60610 (15)	0.0218 (3)	
H13	−0.0316	0.7860	0.6037	0.026*	
C14	0.1304 (2)	0.72054 (5)	0.53793 (14)	0.0185 (3)	
C15	0.3081 (3)	0.79620 (5)	0.44578 (17)	0.0250 (3)	
H15A	0.4393	0.8062	0.3895	0.037*	
H15B	0.3181	0.8126	0.5435	0.037*	
H15C	0.1700	0.8077	0.3929	0.037*	

### Atomic displacement parameters (Å^2^)

**Table:**

	*U*^11^	*U*^22^	*U*^33^	*U*^12^	*U*^13^	*U*^23^
S1	0.0248 (2)	0.01710 (19)	0.01949 (19)	0.00449 (11)	0.00439 (13)	0.00361 (11)
O1	0.0270 (5)	0.0183 (5)	0.0235 (5)	0.0047 (4)	0.0039 (4)	0.0040 (4)
O2	0.0211 (5)	0.0165 (5)	0.0290 (5)	0.0017 (4)	0.0044 (4)	0.0024 (4)
N1	0.0179 (5)	0.0138 (5)	0.0186 (5)	0.0023 (4)	0.0007 (4)	0.0008 (4)
N2	0.0173 (5)	0.0167 (5)	0.0180 (5)	0.0011 (4)	0.0004 (4)	0.0010 (4)
C1	0.0177 (6)	0.0159 (6)	0.0190 (6)	−0.0019 (5)	−0.0008 (5)	−0.0014 (5)
C2	0.0175 (6)	0.0186 (6)	0.0207 (6)	−0.0025 (5)	−0.0018 (5)	0.0000 (5)
C3	0.0170 (6)	0.0200 (7)	0.0281 (7)	0.0000 (5)	−0.0026 (5)	−0.0029 (5)
C4	0.0199 (6)	0.0226 (7)	0.0287 (7)	−0.0019 (5)	0.0051 (5)	−0.0067 (6)
C5	0.0288 (7)	0.0197 (6)	0.0209 (6)	−0.0029 (5)	0.0048 (5)	−0.0008 (5)
C6	0.0245 (7)	0.0174 (6)	0.0210 (6)	0.0001 (5)	0.0003 (5)	0.0009 (5)
C7	0.0180 (6)	0.0181 (6)	0.0172 (6)	−0.0001 (5)	−0.0023 (5)	−0.0003 (5)
C8	0.0158 (6)	0.0161 (6)	0.0174 (6)	−0.0004 (5)	−0.0025 (5)	−0.0009 (5)
C9	0.0170 (6)	0.0178 (6)	0.0168 (6)	0.0031 (5)	−0.0034 (5)	−0.0014 (5)
C10	0.0179 (6)	0.0185 (6)	0.0206 (6)	−0.0002 (5)	−0.0026 (5)	−0.0010 (5)
C11	0.0177 (6)	0.0236 (7)	0.0227 (6)	−0.0003 (5)	−0.0002 (5)	−0.0006 (5)
C12	0.0193 (6)	0.0236 (7)	0.0253 (7)	0.0047 (5)	0.0007 (5)	−0.0013 (5)
C13	0.0223 (7)	0.0170 (6)	0.0260 (7)	0.0034 (5)	−0.0012 (5)	−0.0004 (5)
C14	0.0172 (6)	0.0185 (6)	0.0196 (6)	0.0006 (5)	−0.0026 (5)	0.0015 (5)
C15	0.0279 (7)	0.0166 (7)	0.0307 (7)	−0.0013 (5)	0.0048 (6)	0.0015 (6)

### Geometric parameters (Å, º)

**Table:**

S1—C8	1.6786 (13)	C4—H4	0.9500
O1—C7	1.2257 (16)	C5—C6	1.3862 (19)
O2—C14	1.3582 (16)	C5—H5	0.9500
O2—C15	1.4306 (16)	C6—H6	0.9500
N1—C7	1.3817 (17)	C9—C10	1.3865 (18)
N1—C8	1.3931 (16)	C9—C14	1.4147 (18)
N1—H1n	0.902 (18)	C10—C11	1.3925 (18)
N2—C8	1.3322 (17)	C10—H10	0.9500
N2—C9	1.4140 (16)	C11—C12	1.388 (2)
N2—H2n	0.90 (2)	C11—H11	0.9500
C1—C6	1.3961 (18)	C12—C13	1.391 (2)
C1—C2	1.3969 (18)	C12—H12	0.9500
C1—C7	1.4893 (18)	C13—C14	1.3893 (18)
C2—C3	1.3921 (18)	C13—H13	0.9500
C2—H2	0.9500	C15—H15A	0.9800
C3—C4	1.390 (2)	C15—H15B	0.9800
C3—H3	0.9500	C15—H15C	0.9800
C4—C5	1.390 (2)		
			
C14—O2—C15	116.92 (10)	N2—C8—N1	115.49 (11)
C7—N1—C8	128.10 (11)	N2—C8—S1	126.89 (10)
C7—N1—H1n	116.5 (12)	N1—C8—S1	117.61 (9)
C8—N1—H1n	115.2 (12)	C10—C9—N2	125.27 (12)
C8—N2—C9	129.27 (11)	C10—C9—C14	119.48 (12)
C8—N2—H2n	114.2 (12)	N2—C9—C14	115.12 (11)
C9—N2—H2n	116.4 (12)	C9—C10—C11	120.27 (12)
C6—C1—C2	119.63 (12)	C9—C10—H10	119.9
C6—C1—C7	117.51 (12)	C11—C10—H10	119.9
C2—C1—C7	122.80 (12)	C12—C11—C10	120.18 (13)
C3—C2—C1	119.93 (13)	C12—C11—H11	119.9
C3—C2—H2	120.0	C10—C11—H11	119.9
C1—C2—H2	120.0	C11—C12—C13	120.18 (12)
C4—C3—C2	120.04 (13)	C11—C12—H12	119.9
C4—C3—H3	120.0	C13—C12—H12	119.9
C2—C3—H3	120.0	C14—C13—C12	120.05 (13)
C3—C4—C5	120.12 (13)	C14—C13—H13	120.0
C3—C4—H4	119.9	C12—C13—H13	120.0
C5—C4—H4	119.9	O2—C14—C13	125.55 (12)
C6—C5—C4	120.08 (13)	O2—C14—C9	114.64 (11)
C6—C5—H5	120.0	C13—C14—C9	119.80 (12)
C4—C5—H5	120.0	O2—C15—H15A	109.5
C5—C6—C1	120.20 (13)	O2—C15—H15B	109.5
C5—C6—H6	119.9	H15A—C15—H15B	109.5
C1—C6—H6	119.9	O2—C15—H15C	109.5
O1—C7—N1	122.62 (12)	H15A—C15—H15C	109.5
O1—C7—C1	121.41 (12)	H15B—C15—H15C	109.5
N1—C7—C1	115.96 (11)		
			
C6—C1—C2—C3	−0.12 (19)	C7—N1—C8—S1	−174.57 (10)
C7—C1—C2—C3	−177.16 (12)	C8—N2—C9—C10	−33.1 (2)
C1—C2—C3—C4	0.15 (19)	C8—N2—C9—C14	151.20 (13)
C2—C3—C4—C5	−0.3 (2)	N2—C9—C10—C11	−177.34 (12)
C3—C4—C5—C6	0.4 (2)	C14—C9—C10—C11	−1.80 (18)
C4—C5—C6—C1	−0.4 (2)	C9—C10—C11—C12	−0.1 (2)
C2—C1—C6—C5	0.2 (2)	C10—C11—C12—C13	1.4 (2)
C7—C1—C6—C5	177.44 (12)	C11—C12—C13—C14	−0.7 (2)
C8—N1—C7—O1	−2.4 (2)	C15—O2—C14—C13	−3.32 (19)
C8—N1—C7—C1	177.36 (12)	C15—O2—C14—C9	176.66 (12)
C6—C1—C7—O1	−24.61 (19)	C12—C13—C14—O2	178.76 (12)
C2—C1—C7—O1	152.49 (13)	C12—C13—C14—C9	−1.23 (19)
C6—C1—C7—N1	155.68 (12)	C10—C9—C14—O2	−177.52 (11)
C2—C1—C7—N1	−27.22 (18)	N2—C9—C14—O2	−1.55 (16)
C9—N2—C8—N1	−179.50 (11)	C10—C9—C14—C13	2.46 (19)
C9—N2—C8—S1	−0.8 (2)	N2—C9—C14—C13	178.44 (11)
C7—N1—C8—N2	4.25 (19)		

### Hydrogen-bond geometry (Å, º)

Cg1 is the centroid of the C9–C14 benzene ring.

**Table:**

*D*—H···*A*	*D*—H	H···*A*	*D*···*A*	*D*—H···*A*
N2—H2*n*···O1	0.905 (18)	1.867 (18)	2.6316 (15)	141.0 (16)
C10—H10···S1	0.95	2.68	3.2241 (13)	117
N2—H2*n*···O2	0.90 (2)	2.231 (19)	2.5819 (15)	102.5 (14)
N1—H1*n*···S1^i^	0.902 (18)	2.636 (18)	3.4976 (12)	160.1 (15)
C15—H15*B*···O1^ii^	0.98	2.57	3.4273 (19)	146
C15—H15*C*···*Cg*1^iii^	0.98	2.81	3.6248 (17)	141

Symmetry codes: (i) −*x*+1, −*y*+1, −*z*+1; (ii) *x*, −*y*+3/2, *z*+1/2; (iii) *x*, −*y*+3/2, *z*−1/2.
